# Characterization of a Bacterial Symbiont *Asaia* sp. in the White-Backed Planthopper, *Sogatella furcifera*, and Its Effects on Host Fitness

**DOI:** 10.3389/fmicb.2019.02179

**Published:** 2019-09-18

**Authors:** Fei Li, Hongxia Hua, Asad Ali, Maolin Hou

**Affiliations:** ^1^State Key Laboratory for Biology of Plant Diseases and Insect Pests, Institute of Plant Protection, Chinese Academy of Agricultural Sciences, Beijing, China; ^2^College of Plant Science and Technology, Huazhong Agricultural University, Wuhan, China; ^3^Department of Agriculture, Abdul Wali Khan University Mardan, Mardan, Pakistan; ^4^Scientific Observing and Experimental Station of Crop Pests in Guilin, Ministry of Agriculture, Guilin, China; ^5^Southern Regional Collaborative Innovation Center for Grain and Oil Crops in China, Changsha, China

**Keywords:** *Sogatella furcifera*, bacterial symbiont, *Asaia*, infection, host fitness

## Abstract

The white-backed planthopper (WBPH), *Sogatella furcifera* Horváth (Hemiptera: Delphacidae), is an economically significant rice insect pest that harbors a primary fungal yeast-like symbiont (YLS), and some secondary bacterial symbionts like *Wolbachia* and *Cardinium*. In the present study, an additional bacterial symbiont in WBPH was characterized. Phylogenetic analysis employing the 16S rRNA gene showed a bacterium closely related to *Asaia* of *Nilaparvata lugens* and *Nysius expressus*, and *Asaia krungthepensis*. TEM observation of the bacterium showed the typical morphology of *Asaia* sp. with signature filamentous structures in the nucleoid region. These results indicate that the bacterium belongs to *Asaia*. The *Asaia* bacterium was detected in all the tested individual adults and tissues of the laboratory WBPH population but showed varying infection rates (ca 45%) in the field collected WBPH populations. Quantitative PCR analysis revealed that *Asaia* sp. were significantly more abundant in WBPH females than males, and mainly distributed in the guts, fatty bodies, and salivary glands. *Asaia*-infected WBPH were of shorter nymphal duration and heavier adult weight than *Asaia*-free WBPH, while *Asaia*-free WBPH comparatively fed more, indicating that *Asaia* plays a role in improving WBPH fitness through involvement in host’s nutrient supply.

## Introduction

Insects are colonized by a complex microbial population in a symbiotic relationship (Dillon and Dillon, 2004; Moran et al., 2008). These associations have been formed between symbionts and host insects during long evolutionary processes that are generally categorized as: mutualism, commensalism, and parasitism (Werren et al., 2008; Frago et al., 2012). Some of these symbionts help in important physiological functions for their host insects in many ways. As per the significance of symbionts to the host, symbionts are divided into primary and secondary symbiont (Moran, 2001). The primary symbionts provide nutritional supplements for their hosts such as essential amino acids (Sloan and Moran, 2012), while some secondary symbionts manipulate host reproduction (Werren et al., 2008), or play beneficial roles such as protecting host from parasites and pathogens (Scarborough et al., 2005), increasing heat resistance of host (Dunbar et al., 2007), facilitating host’s digestion of food (Pais et al., 2008), and enhancing host’s insecticide resistance (Tsuchida et al., 2011).

*Asaia* sp. belongs to family Acetobacteriaceae and is an acetic acid bacterium (AAB) (Yamada et al., 2000). Acetic acid bacteria oxidize wine and sugar to acetic acid, except the genus *Asaia* (Crotti et al., 2010). Interestingly, *Asaia* have been reported to be associated with insects that feed on sugar-based diets, particularly those in the order Diptera (Favia et al., 2007; Crotti et al., 2009), Hymenoptera (Kautz et al., 2013; Good et al., 2014), Lepidoptera (Robinson et al., 2010), and Hemiptera (Crotti et al., 2009; Gonella et al., 2012). *Asaia* was first observed by Favia et al. (2007) in the salivary glands, guts and reproductive organs of *Anopheles stephensi*, dominating the microbiota of the mosquito. *Asaia* cells are typically characterized by the signature filamentous structures in the nucleoid region (Favia et al., 2007). Generally, *Asaia* is vertically transmitted from mother to the offspring by an egg smearing mechanism in *A. gambiae* (Crotti et al., 2009), but can also be transmitted to the progeny via venereal transfer during mating in *A. stephensi* (Damiani et al., 2008). *Asaia* isolated from *A. stephensi* and *Aedes aegypti* was able to colonize *Scaphoideus titanus* (Crotti et al., 2009) and transmitted horizontally via mating and co-feeding (Gonella et al., 2012). *Asaia* plays a pivotal role in the growth and reproduction of its hosts. For example, *Asaia* promotes the development of host larvae in *A. stephensi* and *A. gambiae* (Chouaia et al., 2012; Mitraka et al., 2013). *Asaia* co-existing with plasmodium parasites in the guts and salivary glands of *A. stephensi* play an immunity regulatory role through activating the expression of host antimicrobial peptides without inhibiting itself, leading to the possibility of introducing *Asaia* as a strong contender for malaria vector control via paratransgenesis technology (Favia et al., 2007; Damiani et al., 2010; Capone et al., 2013; Rami et al., 2018). Recently, *Asaia* were detected in the small brown planthopper (SBPH, *Laodelphax striatellus*) and the brown planthopper (BPH, *Nilaparvata lugens*) through 16S rDNA high-throughput sequencing technology (Li et al., 2017; Zhang et al., 2019).

The white-backed planthopper (WBPH), *Sogatella furcifera* Horváth, has been reported to harbor the primary fungal yeast-like symbiont (YLS) (Noda et al., 1995) and two secondary bacterial symbionts *Wolbachia* and *Cardinium* (Noda et al., 2001; Nakamura et al., 2012; Zhang et al., 2012a). The YLS makes available sterols to WBPH and also contributes in the nitrogen cycle (Noda and Koizumi, 2003). *Wolbachia* and *Cardinium* function to regulate WBPH reproduction (Noda et al., 2001; Nakamura et al., 2012; Zhang et al., 2012a). It is further determined that *Cardinium* helps to accelerate WBPH nymphal development (Zhang et al., 2012b).

In a previous study, we determined the presence and tissue localization of *Asaia* in WBPH by 16S rDNA high-throughput sequencing and fluorescence *in situ* hybridization (Li et al., 2019). To further characterize the bacterial symbiont *Asaia* sp. in WBPH and its functional association with WBPH, in this study, we detected *Asaia* in WBPH through diagnostic PCR and morphological observation by transmission electron microscopy (TEM), performed phylogenetic analysis by cloning, and sequencing the 16S rDNA of *Asaia*, determined *Asaia* densities in WBPH by quantitative PCR, and measured *Asaia* effects on WBPH fitness.

## Materials and Methods

### White-Backed Planthopper Populations

A laboratory WBPH population (Lab population) originated from a colony collected in 2014 from rice fields in Xing’an (25°36′18″ N, 110°42′16″ E), China, was maintained using caged rice seedlings (var. Taichung Native 1, TN1) for 27 generations in an insectary (27 ± 5°C, relative humidity 80 ± 5%, and a photoperiod of 14 L: 10 D). Three additional WBPH populations (LS population, WH population, and CS population) were collected from the fields in 2016 (Supplementary Table S1). WBPH adults are characterized by a distinct yellowish-white pronotum and mesonotum and can be easily discriminated from adults of other planthopper species. The field-collected insects were kept initially in 95% alcohol and subsequently stored at −80°C until DNA extraction and were used only in detecting infection rates of symbiotic bacteria.

### Detection of *Asaia* in WBPH

Presence of *Asaia* in WBPH was previously determined by 16S rDNA high-throughput sequencing and fluorescence *in situ* hybridization (Li et al., 2019). Diagnostic PCR were used to further detect the presence of *Asaia* in individual adults of the four WBPH populations and in tissues and individual offspring adults of the Lab population using the *Asaia*-specific primers. The adult tissues of the Lab population were obtained by dissecting the adults in a drop of sterile 0.01 M PBS buffer (Ph 7.2) under a stereo microscope. The offspring adults (F1 generation) were obtained by pairing parents in glass tubes (180 mm in height and 30 mm in diameter) with 3-leaf rice seedling and rearing the resulting newly hatched nymphs individually in new glass tubes with rice seedlings until they became adults. Total DNA was extracted from individual adults and the tissues using the Wizard^®^ Genomic DNA Purification Kit (Promega, United States), according to the manufacturer’s protocols. The *Asaia*-specific primers Asafor and Asarev (Table 1) were used to amplify a sequence of 181 bp from the 16S rRNA gene of *Asaia* (Favia et al., 2007). The PCR conditions were: 1 cycle of 94°C for 5 min; 35 cycles of 94°C for 30 s, 62°C for 30 s, and 72°C for 30 s; and a final extension of 72°C for 10 min. Sterile water was included in all PCRs as a negative control and the DNA sample of WBPH verified by cloning and sequencing was used as a positive control.

**TABLE 1:** The specific primers used in diagnostic PCR.

**Symbiont**	**Target gene**	**Primer**	**Nucleotide sequence (5′ to 3′)**	**References**
*Wolbachia*	*wsp*	*wsp*-81F	TGGTCCAATAAGTGATGAAGAAAC	Zhou et al., 1998
		*wsp*-691R	AAAAATTAAACGCTACTCCA	
*Cardinium*	16S rDNA	CLO-F	GCGGTGTAAAATGAGCGTG	Weeks et al., 2003
		CLO-R1	ACCTCTTCTTAACTCAAGCCT	
*Asaia*	16S rDNA	Asafor	GCGCGTAGGCGGTTTACAC	Favia et al., 2007
		Asarev	AGCGTCAGTAATGAGCCAGGTT	
YLS	18S rDNA	NF	GCGGTAATTCCAGCTCCAA	Hou et al., 2013
		NR	CCCCGACTGTCCCTATTAATC	

The presence of *Asaia* in WBPH was further morphologically detected using transmission electron microscope (TEM). Adult guts of the Lab population were dissected. After the gut samples were fixed in 2% glutaraldehyde overnight at 4°C and then washed with 0.1 M PBS buffer (pH 7.2) four times (30 min each time), they were postfixed in 1% OsO_4_ in 0.1 M sodium cacodylate buffer (pH 7.2) for 2 h at 4°C and washed with 0.1 M PBS buffer (pH 7.2) two times (15 min each time). Thereafter, the samples were dehydrated through a graded ethanol series (30, 50, 70, 80, 90, 95, and 100%) for 10 min at each concentration, and finally through acetone for three 10-min times. The prepared samples were embedded in 618 epoxy resin (Sigma, United States) and polymerized at 37°C for 12 h, 45°C for 12 h, and 60°C for 48 h, and then sliced into ultrathin sections (60 nm) by a Leica EM UC6 ultrathin slicer (Leica, Germany) and stained with uranyl acetate. The final sample sections were examined under a Hitachi-7500 TEM (Hitachi, Japan) (Crotti et al., 2009).

### Molecular Phylogenetic Analysis of *Asaia* in WBPH

The 16S rRNA gene was used to identify *Asaia* in the four populations. Total DNA was extracted from individual adults of the four populations as above. The bacterial 16S rRNA gene was amplified using the universal primers 27F (5′-AGAGTTTGATCCTGGCTCAG-3′) and 1492R (5′-ACGGTTACCTTGTTACGACTT-3′) (Weisburg et al., 1991). The PCR analyses were conducted with Taq polymerase (Takara, Japan) in an ABI 9700 thermocycler (Thermo Fisher Scientific, United States). The PCR conditions were: 1 cycle of 94°C for 3 min; 35 cycles of 94°C for 30 s, 52°C for 30 s, and 72°C for 2 min; and a final extension of 72°C for 10 min. Amplified fragments were purified by the AxyPrep^TM^ DNA Gel Extraction Kit, according to the manufacturer’s protocols (Corning, United States), and the PCR products with the expected size were cloned into the pMD-19T plasmid vector (Takara, Japan) and sequenced in an ABI 3730XL DNA analyzer. The 16S rRNA gene sequences of *Asaia* were registered in GenBank under accession numbers of MK814862, MK811206, MK811207 (Supplementary Table S1), and MK598732 for the LS, WH, CS, and Lab populations, respectively.

A phylogenetic tree was constructed using MEGA v6.0 (Tamura et al., 2013). DNA sequence similarities among *Asaia* species were investigated using the BLAST search tool^1^. A multiple alignment of nucleotide sequences was performed using the program package Clustal W with the default parameters (Tamura et al., 2013). The final alignment was manually inspected and corrected (deposited under Supplementary Materials). The phylogenetic tree was constructed using the maximum likelihood (ML) method with HKY + G model. Bootstrap analysis of 1,000 replicates was used to deduce confidence levels.

### *Asaia* Densities in Relation to WBPH Stages and Tissues

*Asaia* densities were quantified by real-time fluorescence quantitative PCR (qPCR) in various tissues and developmental stages of the Lab population. Twenty newly emerged WBPH adults were dissected as above to obtain a sample of each of the salivary glands, guts, reproductive organs, and fat bodies for qPCR. *Asaia* densities were also measured in different developmental stages, i.e., nymphs of five instars (20, 10, 5, 5, and 5 insects pooled per sample, respectively) and female and male adults of 8 age groups (0, 2, 4, 6, 8, 10, 12, and 14 d post emergence) (5 insects pooled per sample).

The total DNA was extracted as per the method described above. The copy numbers of the 16S RNA gene of *Asaia* were quantified using specific primers Asafor and Asarev (Table 2). qPCR analyses were conducted with SYBR^®^ Premix Ex Taq^TM^ II (Takara, Japan) in ABI 7500 Real-Time System (Thermo Fisher Scientific, United States). The PCR temperature profile was 2 min at 95°C 40 cycles of 5 s at 95°C and 34 s at 60°C. The qPCR analyses were biologically performed 3 times. The number of 16S rRNA gene copies of *Asaia* in the individual tissues and insects was calculated using absolute quantification analysis. A serial of dilutions (10^2^, 10^3^, 10^4^, 10^5^, 10^6^, 10^7^, and 10^8^) of the standard plasmids, containing the sequence of 181 bp from the 16S rRNA gene of *Asaia*, was used as PCR templates for the establishment of a standard curve.

**TABLE 2:** The specific primers of quantitative PCR to quantify the density of symbionts in WBPH.

**Symbiont**	**Target gene**	**Primer**	**Primer sequence(5′ to 3′)**	**References**
*Wolbachia*	16S rDNA	INTF2	AGTCATCATGGCCTTTATGGA	Sakamoto et al., 2006
		INTR2	TCATGTACTCGAGTTGCAGAGT	
*Cardinium*	16S rDNA	CLOF	CCAAGGCTATGGGTAGG	Zhang et al., 2012a
		CLOR	CATGGCTTCAGGCTTG	
*Asaia*	16S rDNA	Asafor	GCGCGTAGGCGGTTTACA	Favia et al., 2007
		Asarev	AGCGTCAGTAATGAGCCAGGTT	
YLS	18S rDNA	Noda-F	TCCCTCTGTGGAACCCCAT	Cao et al., 2015
		Noda-R	GGCGGTCCTAGAAACCAACA	

### *Asaia* Effects on WBPH Fitness

To verify the effects of *Asaia* on WBPH fitness, an *Asaia*-free strain (A^–^ strain) was established via oral treatment of WBPH with tetracycline hydrochloride (Amresco, United States). In a glass beaker (180 mm in height and 100 mm in diameter), 150 ml 0.25 mg/ml tetracycline hydrochloride was added to a cotton pad lined on the bottom, and 100 rice seeds (TN1) were seeded on the cotton pad for germination in the insectary. After 7 days, 100 newly hatched nymphs of the Lab population (F0 generation) that were infectious with YLS, *Wolbachia*, *Cardinium* and *Asaia* (A^+^ strain) were introduced into the beaker and allowed to feed ad lib in the insectary. Twelve days later, the resulting F0 adults were paired in glass tubes with an antibiotic-free 3-leaf rice seedling for oviposition. The hatched nymphs were left to develop on antibiotic-free rice seedlings, the resulting newly emerged F1 adults were paired for oviposition for 7 days and then individually detected for presence of symbionts (YLS, *Wolbachia*, *Cardinium* and *Asaia*) using diagnostic PCR and the specific primers (Table 1). If both F1 parent adults were negative with *Asaia* while positive with YLS, *Wolbachia* and *Cardinium*, their offsprings (A^–^ strain) were reared on antibiotic-free rice plants for another 9 generations to eliminate possible side effects of the antibiotic treatment (Noda et al., 2001) before they were used in fitness measurements. The A^+^ and A^–^ strains were periodically subjected to qPCR for quantification of YLS, *Wolbachia*, *Cardinium*, and *Asaia* to make sure that the two obtained strains differed only in *Asaia*.

To measure *Asaia* effects on WBPH nymphal duration and adult weight, 100 newly hatched nymphs (<12 h) of each of the A^+^ and A^–^ strains were randomly collected and individually reared on 15 3-leaf rice seedlings in the glass tube in the insectary. Nymphal duration was examined to 30 nymphs and weight was measured to 30 newly emerged male and female adults from each strain.

Newly emerged WBPH adults (<12 h) of each of the A^+^ and A^–^ strains were randomly selected and paired in the glass tube with 15 3-leaf rice seedlings for test of fecundity and longevity. Upon adult death, fecundity per female (number of hatched nymphs plus number of eggs remaining in the seedlings) and adult longevity were recorded. The tests were run for 30 adult pairs for each strain.

Feeding amount represented by honeydew excretion was measured using a parafilm sachet method (Pathak et al., 1982). Three newly emerged female adults of each of the A^+^ and A^–^ strains starved for 1 h were aspirated into a parafilm sachet (5 cm × 5 cm) attached to the stem of a tillering rice plant in the insectary. After 24 h, the insects were removed and each of the parafilm sachets was weighed immediately and, after removal of the honeydew, were weighed again using a balance (XP205, Mettler Toledo, Switzerland). The net weight of honeydew excretion was calculated. The tests were repeated for 30 times for each WBPH strain.

### Statistical Analysis

All the data of symbiotic bacteria densities were converted by logarithm before statistical analysis. *Asaia* densities in tissues and nymphal instars of the Lab population WBPH were analyzed by one-way analysis of variance (ANOVA), means were separated using a Tukey test when equal variance was assumed or Games-Howell test when equal variance was not assumed. Two-way analysis of variance (ANOVA) was performed to analyze for significant influence of age and sex on densities of *Asaia* in WBPH adults, differences in *Asaia* densities between female and male of different ages were analyzed by independent sample *T* test. The differences in fitness parameters of the A^+^ and A^–^ strains were analyzed by independent sample *T* test. All statistical analyses were performed with IBM SPSS Statistics 25.0 (IBM, United States) at *P* < 0.05.

## Results

### Detection of *Asaia* in WBPH

The presence of *Asaia* sp. in WBPH was verified through detection of the 16S rRNA gene of *Asaia* in individual adults of the four populations and tissues of the Lab population using *Asaia*-specific primers (Table 1). *Asaia* was present in all the individual parental adults and tissues of the Lab population, while the infection rates of both female and male adults of the field-collected populations varied from 36.7 to 63.3% and from 33.3 to 53.3%, respectively, averaging 46.7% (42/90) and 45.6% (41/90) (Table 3). When newly hatched nymphs of the parental insects of the Lab population were reared individually, the *Asaia* infection in the resulting offspring adults was only 30% (36/120) (Table 3).

**TABLE 3:** The infection rates of *Asaia* in WBPH individual adults and tissues of four populations.

**Population**	**Individual insects/tissues**	**Total number of tested samples (Number of positive samples)**
LS	Adults	30♀ (12); 30♂ (16)
WH	Adults	30♀ (19); 30♂ (15)
CS	Adults	30♀ (11); 30♂ (10)
Lab	Parent adults	30♀ (30); 30♂ (30)
	♀ salivary glands	30 (30)
	♀ guts	30 (30)
	♀ reproductive systems	30 (30)
	♂ guts	30 (30)
	♂ reproductive systems	30 (30)
	Offspring adults (F1 generation)	60♀ (19); 60♂ (17)

**FIGURE 1:**
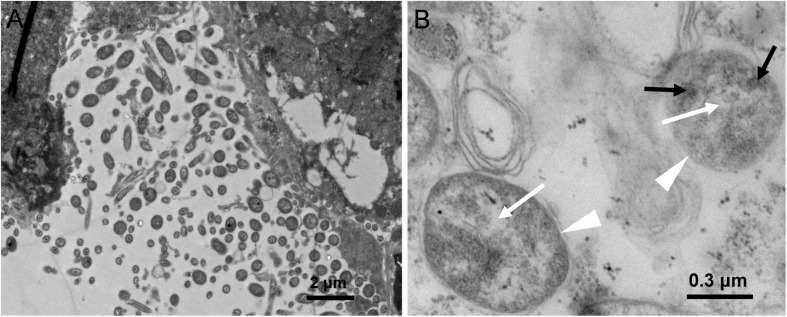
TEM micrographs of an WBPH adult female hindgut. **(A)** Hindgut lumen of female WBPH. **(B)** Detail of panel **A**, showing two bacteria (indicated by white triangle), where black arrowheads indicate enterosomes in the bacterial cytoplasm, and white arrowheads indicate bright filamentous nucleoid region.

Presence of *Asaia* in WBPH was further morphologically detected by TEM in hindgut sections of WBPH. Bacterial cells resembling *Asaia* were observed (Figure 1), confirming the results of PCR analysis. Figure 1A shows a portion of the hindgut of WBPH adults harboring a polymorphic bacterial flora. Some of the bacteria presented the morphological signatures of *Asaia* sp., i.e., bright filamentous nucleoid region and electron-dense cytoplasm microinclusions resembling enterosomes (Figure 1B), previously described for *Asaia* cells in *A. stephensi*, *A. aegypti*, and *S. titanus* (Favia et al., 2007; Crotti et al., 2009). These signatures of bacterial cells suggest the presence of *Asaia* in WBPH.

### Molecular Phylogenetic Analysis of *Asaia* in WBPH

**FIGURE 2:**
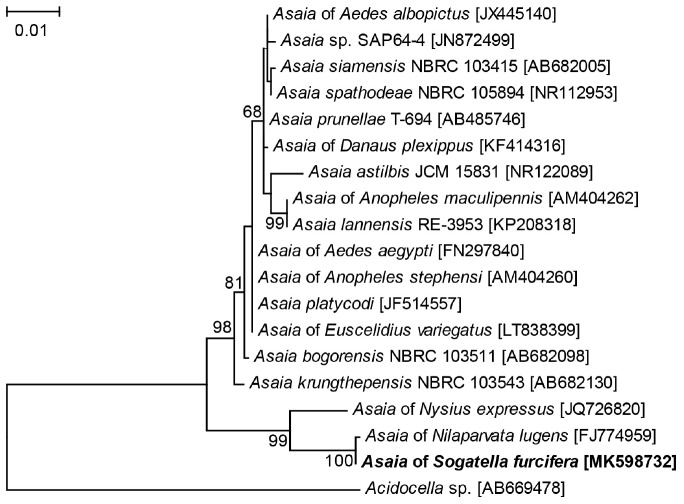
Phylogenetic tree based on *Asaia* 16S rRNA gene sequences. The tree was constructed using HKY + G model for ML method. The names and sequence GenBank accession numbers (in parentheses) are shown. Sequences obtained in this study are shown in bold. Only Bootstrap values >50 are represented. *Acidocella* sp. of the family Acetobacteriaceae is used as outgroup.

Four 1452 bp 16S rRNA gene sequences of *Asaia* were obtained from the four WBPH populations, which were deposited in GenBank. The sequences showed limited variation between the four populations (0–1 nucleotide differences), and the 16S rRNA gene sequence of *Asaia* in the Lab population was used in phylogenetic analysis. According to BLAST analysis, the 16S rRNA sequence of WBPH showed 99.93% similarity to that from *Asaia* of BPH (GenBank no. FJ774959), 97.93% to that from *Asaia* of *Nysius expressus* (GenBank no. JQ726820), and 96.33% to that from *Asaia krungthepensis* (GenBank no. AB682130). Phylogenetic analysis of the 16S rRNA sequences using ML method clustered *Asaia* of WBPH, BPH and *N. expressus* to a single clade that was distinct from the known *Asaia* of mosquitoes and *A. krungthepensis* (Figure 2).

### *Asaia* Densities in Relation to WBPH Stages and Tissues

**FIGURE 3:**
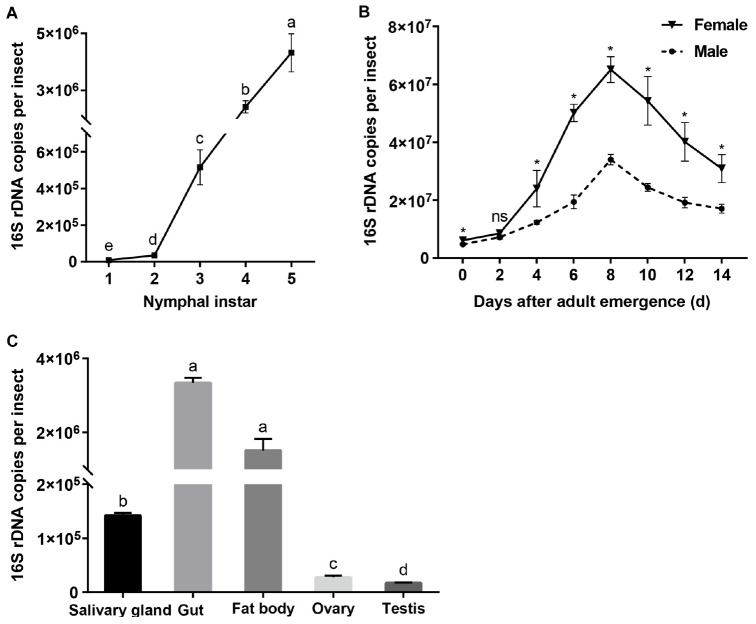
*Asaia* densities in WBPH. **(A)** Nymphs. **(B)** Adults. **(C)** Tissues. The data are expressed as means ± sd. Different letters in panel **A** and panel **C** indicate significant differences based on Tukey test and Games-Howell test at *P* < 0.05, respectively. In panel **B**, ^∗^ indicates significant difference and ns, no significant difference, between female and male of different ages according to independent sample *T* test at *P* < 0.05.

When expressed as the number of 16S rRNA gene copies per insect, *Asaia* densities increased with the nymphal instar (*F* = 1033.081, df = 4,10, *P* < 0.001; Figure 3A), being the lowest in the first instars and increasing significantly as the nymphs developed through the second to the fourth instars (Tukey test, *P* ≤ 0.001). In the adult stage, *Asaia* densities showed a unimodal type change with adult age, peaking in 8 days old females and males (Figure 3B). ANOVA showed that the *Asaia* densities in WBPH adults were significantly affected by both age (*F* = 221.682, df = 7,32, *P* < 0.001) and sex (*F* = 273.767, df = 1,32, *P* < 0.001) and their interaction (*F* = 6.775, df = 7,32, *P* < 0.001). *Asaia* densities in females were significantly higher than those in males with the exception of 2 days old adults (*t* ≥ 3.552, df = 4, *P* ≤ 0.024; Figure 3B). Among the tissues, *Asaia* abundance differed significantly (*F* = 1305.572, df = 4, 10, *P* < 0.001; Figure 3C), being the most in the guts and the fatty bodies, significantly lower in the salivary glands, and still significantly lower in the reproductive organs (ovaries and testes) (Games-Howell test, *P* ≤ 0.023).

### *Asaia* Effects on WBPH Fitness

**FIGURE 4:**
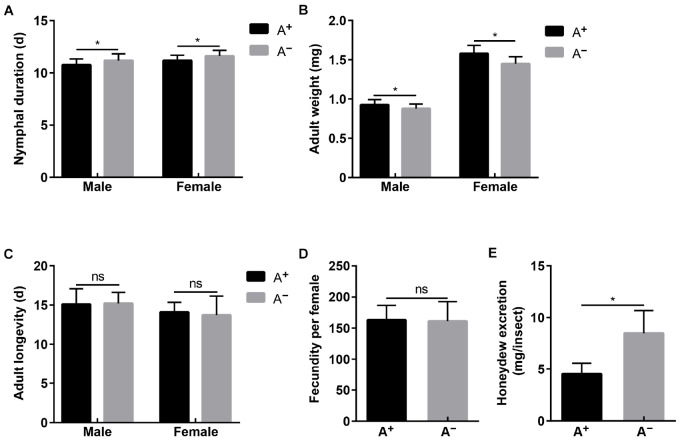
Fitness of A^+^ and A^–^ WBPH strains that differed in *Asaia* densities while showed no differences in densities of YLS, *Wolbachia* and *Cardinium*. **(A)** Nymphal duration. **(B)** Adult longevity. **(C)** Adult weight. **(D)** Fecundity. **(E)** Honeydew excretion. Means and standard deviation are shown. Asterisk indicate significant differences based on independent sample *T* test at *P* < 0.05, while ns indicates no significant difference.

The established A^+^ and A^–^ WBPH strains differed significantly in *Asaia* density (male: *t* = 314.792, df = 4, *P* < 0.001; female: *t* = 766.028, df = 4, *P* < 0.001), while they showed no differences in densities of YLS (male: *t* = 0.918, df = 4, *P* = 0.410; female: *t* = 0.302, df = 4, *P* = 0.778), *Wolbachia* (male: *t* = 0.133, df = 4, *P* = 0.900; female: *t* = 0.413, df = 4, *P* = 0.700), and *Cardinium* (male: *t* = 0.227, df = 4, *P* = 0.832; female: *t* = 0.555, df = 4, *P* = 0.608) (Supplementary Figure S1). With these two strains, *Asaia* effects on WBPH fitness were assessed in terms of nymphal duration, adult weight, fecundity, adult longevity, and feeding amount. Nymphal development of A^–^ strain was delayed 0.41 and 0.44 d in males and females compared with that of A^+^ strain, respectively (male: *t* = 3.016, df = 58, *P* = 0.004; female: *t* = 2.817, df = 58, *P* = 0.007; Figure 4A). Newly emerged adults of A^+^ strain weighed significantly more than adults of A^–^ strain by a difference of 0.05 mg in males (*t* = 3.830, df = 58, *P* < 0.001) and a difference of 0.13 mg in females (*t* = 5.324, df = 58, *P* < 0.001; Figure 4B). However, feeding amount represented by honeydew excretion was significantly greater in A^–^ females (8.50 mg) than in A^+^ females (4.53 mg) (*t* = 8.961, df = 58, *P* < 0.001; Figure 4E). No differences were observed in adult longevity (male: *t* = 0.262, df = 58, *P* = 0.794; female: *t* = 0.734, df = 58, *P* = 0.466; Figure 4C) and fecundity (*t* = 0.261, df = 58, *P* = 0.795; Figure 4D) between A^+^ strain and A^–^ strain.

## Discussion

So far, *Asaia* has been detected in many insects, including rice planthoppers SBPH (Li et al., 2017) and BPH (Zhang et al., 2019). Recently, an *Asaia* sp. was detected in WBPH populations through 16S rDNA high-throughput sequencing (Li et al., 2019). The present study further confirmed the presence of *Asaia* in WBPH via diagnostic PCR using the 16S rRNA gene (Table 3) and TEM observation (Figure 1). *Asaia* was detected in about 45% of adults of the three field collected WBPH populations (Table 3), contrasting to the 100% infection in adult wild populations of *A. gambiae* (Damiani et al., 2010). The reason may be that the *Asaia* in *A. gambiae* is vertically transmitted at 100% (Damiani et al., 2010), while the *Asaia* in WBPH is vertically transmitted at 30% (Table 3). The low vertical transmission of *Asaia* in WBPH is consistent with the low density of *Asaia* found in the ovaries and testes (Figure 3C). Nevertheless, the adult individuals and tissues of the Lab WBPH population were 100% infected with *Asaia* (Table 3), which was consistent with the results in the laboratory population of *A. gambiae* (Damiani et al., 2010). Also, *Asaia* is capable of intraspecies horizontal transmission in *S. titanus* (Gonella et al., 2012) and interspecies cross-colonizing between *Ae. aegypti*, *A. stephensi* and *S. titanus* (Crotti et al., 2009). The 100% infection with *Asaia* in the Lab WBPH population (Table 3) is probably due to high horizontal transmission of *Asaia* in co-feeding WBPH reared in a cage. Therefore, it can be hypothesized that low vertical transmission coupled with high horizontal transmission in co-feeding lead to the discrepancies in *Asaia* infection between the Lab and field collected WBPH populations. In TEM observation, the bacterial cells in WBPH showed the typical morphology of *Asaia* sp. with signature filamentous structures in the nucleoid region (Figure 1B) like the *Asaia* cells in *A. aegypti* and *S. titanus* (Crotti et al., 2009). With these results, it can be ensured that *Asaia* sp. exists in WBPH.

Phylogenetic analysis of 16S rRNA gene sequences clustered the symbiotic bacteria *Asaia* in WBPH, BPH, and *N. expressus* to the same branch that was distinct from the known *Asaia* of mosquitoes and *A. krungthepensis* (Figure 2). According to Tindall et al. (2010), when similarity of a microorganism 16S rRNA gene sequence to all the others is <97%, it is acceptable to classify the microorganism as a new species; and when the similarity is ≤95%, the microorganism may be classified into a new genus. The 16S rRNA gene of *Asaia* in WBPH exhibits 96.33% sequence similarity with that of the previously described *A. krungthepensis*. Therefore, the *Asaia* of WBPH may be a new species different from *A. krungthepensis*.

*Asaia* abundance in WBPH nymphs experienced an exponential increase with the progress of instars (Figure 3A). In *Diaphorina citri* nymphs, *Wolbachia* also showed a similar increase pattern in relation to nymph instars (Dossi et al., 2014). In the adults, *Asaia* abundance underwent a unimodal change, peaking in 8 days old adults (Figure 3B), indicating *Asaia* proliferation was correlated with the reproductively active stage of WBPH. Similar results have been reported for *Buchnera* density in adult aphids (Baumann, 2005; Sakurai et al., 2005). *Asaia* density decreased in the old WBPH adults (Figure 3B); a similar symbiont drop has also been observed in *Acyrthosiphon pisum* (Nishikori et al., 2009) and *Camponotus floridanus* (Ratzka et al., 2013). Interestingly, *Asaia* densities in the newly emerged WBPH adults were not different from those in the old nymphs (Figures 3A,B). *Asaia* densities also responded to the sex, being higher in WBPH females than in males (Figure 3B). Female-biased symbiont densities have also been reported for *Wolbachia* and *Cardinium* in WBPH (Noda et al., 2001; Nakamura et al., 2012; Zhang et al., 2012a). It can be expected that the higher density in female adults may benefit from vertical transmission of symbionts, as suggested for *Portiera* in *Bemisia tabaci* (Bing et al., 2013). In horizontal transmission through-feeding, symbionts gained from diet first reach the gut lumen, after crossing numerous physical and biochemical barriers in the haemocoel, and can finally colonize different tissues (Vallet-Gely et al., 2008). Quantitative PCR data showed that *Asaia* in WBPH largely colonized the salivary glands, guts and fat bodies, and a small amount of *Asaia* was found in the testes and ovaries (Figure 3C), confirming our results suggesting tissue localization of *Asaia* in WBPH (Table 3). A similar tissue specific *Asaia* abundance was reported in *Anopheles* mosquitoes, where *Asaia* acquired through feeding numerously colonized the gut and then spread to other tissues through hemolymph (Favia et al., 2007; Rami et al., 2018). Abundance differences of *Asaia* among/between tissues, sexes, nymphal and adult stages may relate to the biological functions for WBPH, which need to be determined in further studies.

*Asaia* effects on WBPH fitness were assessed using A^+^ and A^–^ WBPH strains. *Asaia* promoted nymphal development (Figure 4A) and increased adult weight (Figure 4B), which corresponds to the rapid increase in *Asaia* density in progressing nymphal instars (Figure 3A), indicating that *Asaia* may play a role in improving WBPH fitness, as *Buchnera* providing essential amino acids for aphids (Baumann, 2005; Sakurai et al., 2005). Our results agree with previous studies in *Anopheles* mosquitoes, where *Asaia* accelerated development of *A. stephensi* (Chouaia et al., 2012) and *A. gambiae* (Mitraka et al., 2013), and increased nymphal weight (Mitraka et al., 2013). In phloem sap feeding Hemiptera insects, the amount of honeydew is used to represent food intake (Pathak et al., 1982). The amount of honeydew secreted by the A^–^ strain almost doubled that of the A^+^ strain (Figure 4E), similar to the result reported in *Aphis fabae* in the absence of secondary symbionts (Schillewaert et al., 2017). The phloem sap of rice is characterized by an imbalance of nutrient components and cannot satisfy the normal growth and developmental needs for planthoppers (Xue et al., 2014). Therefore, we assume that *Asaia* plays a role in supplementing the nutritional needs of WBPH. Thereby, when WBPH are deprived of *Asaia*, the insects compensate lack of nutrients through increasing feeding. Surprisingly, the differences in adult longevity (Figure 4C) and fecundity (Figure 4D) between A^+^ and A^–^ strains were not significant. These results show that, even though *Asaia* is not essential for WBPH survival, it affects the host fitness to some extent probably through involvement in host nutrient supply. Further studies using metagenomics and proteomics techniques are needed to investigate the fundamental mechanism underlying *Asaia* effects on WBPH fitness, especially its nutritional role for the host. It is worth noting that feeding WBPH on tetracycline hydrochloride-treated rice seedlings resulted in A^–^ insects at about 80%, which indicates that *Asaia* may be more susceptible to tetracycline hydrochloride than the other symbionts. *Asaia* is mostly harbored in the alimentary organs, such as the guts and salivary glands, thus making it easily exposed to the antibiotics.

In summary, we detected the presence of *Asaia* in WBPH through TEM observation and diagnostic PCR, characterized its phylogenies, density dynamics, and effects on host fitness. *Asaia* of WBPH, BPH, and *N. expressus* are phylogenetically in a single clade that is distinct from the known *Asaia* of mosquitoes and *A. krungthepensis*. *Asaia* densities increase with WBPH nymphal development, and are greater in gut, fat body and salivary gland, and greater in females than in males. *Asaia* in WBPH functions to accelerate nymphal development and increase adult weight, while reduce feeding. A better understanding of the interactions between host insects and symbionts will provide ample information on the potential of manipulating microbiota to control insect pests. Further studies are needed to explore the roles of *Asaia* at the nutritional and molecular level, and the details of its horizontal transmission in WBPH.

## Data Availability Statement

The datasets generated for this study can be found in the MK814862, MK811206, MK811207, and MK598732.

## Author Contributions

FL, HH, and MH conceived and designed the experiments. FL performed the experiments. FL and MH analyzed the data. FL, AA, and MH wrote the manuscript. All the authors read and approved the final version of the manuscript.

## Conflict of Interest

The authors declare that the research was conducted in the absence of any commercial or financial relationships that could be construed as a potential conflict of interest.

## Footnotes

1 http://blast.ncbi.nlm.nih.gov/Blast.cgi
