# Supplementary material for: Characterization of a Bacterial Symbiont Asaia sp. in the White-Backed Planthopper, Sogatella furcifera, and Its Effects on Host Fitness
Source: Front Microbiol. 2019 Sep 18;10:2179. doi: 10.3389/fmicb.2019.02179 (PMC6759652; doi:10.3389/fmicb.2019.02179)
Supplement: Supplementary file 1 [file Table_1.DOCX]

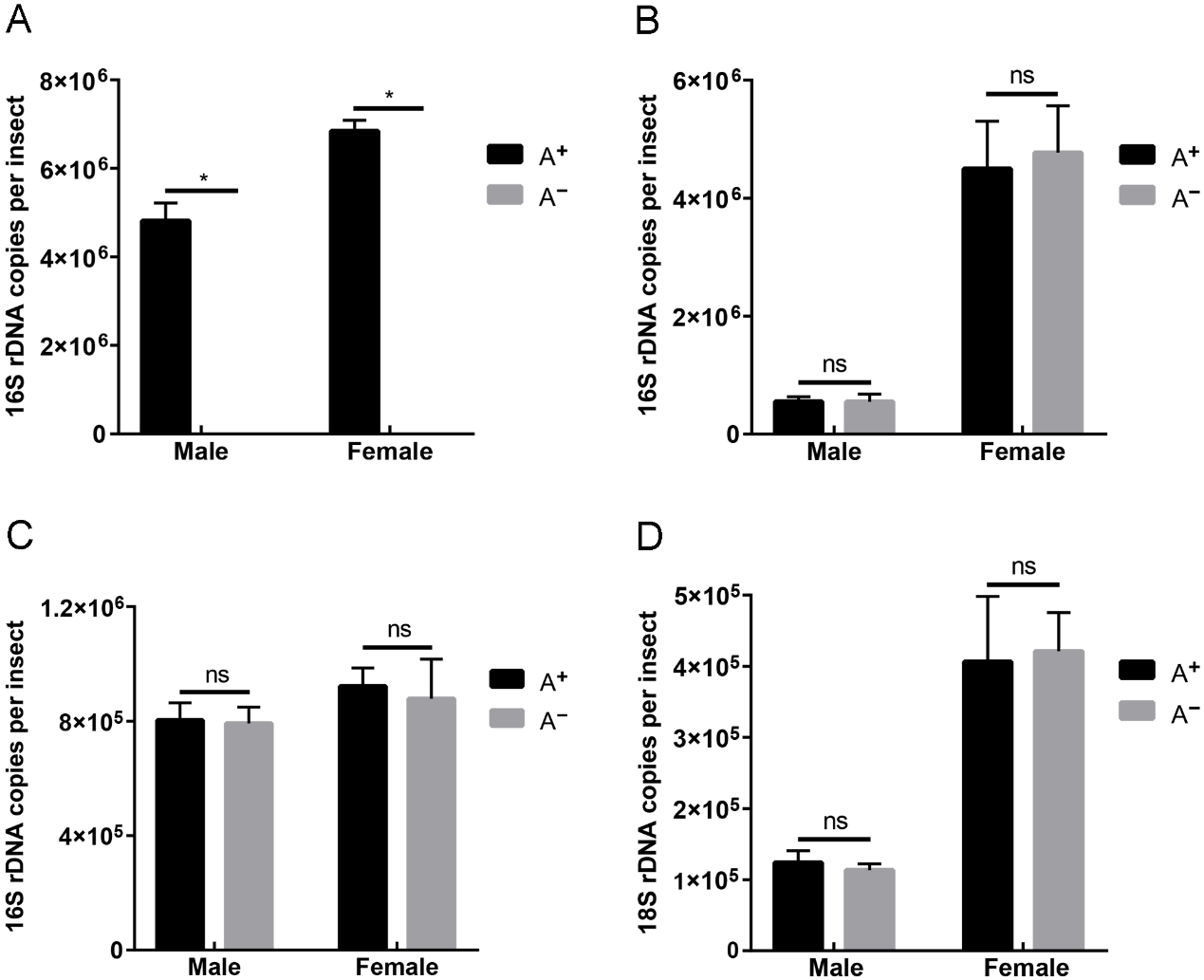


**FIGURE S1 |** Densities of symbionts in A^+^ and A^−^ strain WBPH. (A) *Asaia*. (B) *Wolbachia*. (C) *Cardinium*. (D) YLS. A^+^ strain was *Asaia*-free WBPH, A^−^ strain was *Asaia*-infected WBPH. Bar represents standard errors of means and asterisk indicate significant differences based on independent sample T test at *P* < 0.05, while ns indicates no significant difference.
