# Supplementary material for: Characterization of a Bacterial Symbiont Asaia sp. in the White-Backed Planthopper, Sogatella furcifera, and Its Effects on Host Fitness
Source: Front Microbiol. 2019 Sep 18;10:2179. doi: 10.3389/fmicb.2019.02179 (PMC6759652; doi:10.3389/fmicb.2019.02179)
Supplement: Supplementary file 2 [file Table_2.DOCX]

**TABLE S1 |** Detection of *Asaia* in WBPH populations

| WBPH population | Locality in China | Latitude | Longitude | Collection date | GenBank accession no. of bacterium 16S rRNA gene |
| --- | --- | --- | --- | --- | --- |
| LS | Lingshui, Hainan | 18°32′28′′N | 110°4′15′′E | 2016/6/3 | MK814862 |
| WH | Wuhan, Hubei | 30°28′43′′N | 114°21′2′′E | 2016/6/15 | MK811206 |
| CS | Changsha, Hunan | 28°7′40′′N | 113°20′1′′E | 2017/6/30 | MK811207 |
